# Early outcomes with a flexible ECAP based closed loop using multiplexed spinal cord stimulation waveforms—single-arm study with in-clinic randomized crossover testing

**DOI:** 10.1093/pm/pnaf058

**Published:** 2025-05-16

**Authors:** Vahid Mohabbati, Richard Sullivan, James Yu, Peter Georgius, Charles D Brooker, Malgorzata Siorek, Nancy L McClelland, Filippo Coletti, Xiaoxi Sun, Abi Franke, Marc A Russo

**Affiliations:** Sydney Pain Research Center, Wahroonga, NSW 2076, Australia; Precision Brain Spine & Pain Centre, Kew, VIC 3101, Australia; Australian Medical Research, Hurstville, NSW 2220, Australia; Sunshine Coast Clinical Research, Noosa Heads, QLD 4567, Australia; Royal North Shore Hospital, St. Leonards, NSW 2065, Australia; Medtronic Neuromodulation, Minneapolis, MN 55432, United States; Medtronic Neuromodulation, Minneapolis, MN 55432, United States; MCRS, Study and Scientific Solutions, Rome, RM 00165, Italy; Medtronic Neuromodulation, Minneapolis, MN 55432, United States; Medtronic Neuromodulation, Minneapolis, MN 55432, United States; Genesis Research Services, Broadmeadow, NSW 2292, Australia

## Abstract

**Background:**

Spinal cord stimulation (SCS) systems that deliver fixed amplitudes expose target tissue to varying electrical fields due to the changing lead-to-cord distance accompanying postural shifts and other body movements. Inconsistent stimulation results in periods of overstimulation or under-stimulation perceived by patients as discomfort or potentially inadequate pain relief. CL-SCS may be applied to provide a comfortable level of perception down to imperceptible stimulation, commonly preferred in higher frequency and multiplexed programming. Here we report outcomes from a study evaluating a closed-loop (CL) SCS system that uses spinal evoked compound action potentials to adjust stimulation.

**Methods:**

This ongoing study combines the evaluation of pain-related outcomes (for ≤24 months) with in-clinic randomized, crossover testing of CL performance.

**Results:**

Sixty subjects have been implanted with the CL-SCS system, and 54 subjects have completed the 3-month visit. Sixty percent preferred below-perception waveforms for therapy during at-home use. The study successfully met its primary endpoint with 89.3% of subjects in the Primary Analysis Set (*n* = 28) reporting reduction in overstimulation with CL-SCS relative to OL-SCS at 1 month (*P* < .001; binomial exact test); at 3 months, 86% of subjects with low-back/leg pain (*n* = 51); and all 3 with upper limb pain reported ≥50% reduction in pain, relative to baseline.

**Conclusions:**

The data presented here support the performance of a flexible CL-SCS system that can deliver a variety of waveforms, with amplitude programmed to patient comfort and automatically adjusted up to 50 times per second, to improve the consistency of therapy experience.

**Registration:**

This study is registered on ClinicalTrials.gov number NCT05177354 https://clinicaltrials.gov/search? term=NCT05177354

## Introduction

Spinal cord stimulation (SCS) is an important and cost-effective therapy for the treatment of chronic neuropathic pain refractory to more conservative treatement.^1–3^ Most SCS systems, whether they deliver conventional paresthesia-based therapy or modern (eg, high frequency, burst, differential target multiplexed) waveforms are programmed with fixed-amplitude (or open-loop [OL]) stimulation to achieve analgesia. The stimulation amplitude is determined during in-clinic programming, with patient feedback, and the settings may be further adjusted over multiple visits or by the patient at home. Several SCS systems provide automatically adjusted (adaptive or closed-loop [CL]) stimulation amplitude in response to a detected signal, reducing the need for a patient to make manual adjustments.

Analogous to drug delivery, the term “dose” has been used to describe the total amount of electrical energy delivered by the stimulation in a given period of time. A dose too low is proposed as ineffective and too high as resulting in potentially treatment-limiting side effects; with an ideal dose being both comfortable and effective. The delivery of stimulation from fixed-amplitude SCS systems can lead to inconsistencies in the electrical energy reaching the spinal cord. This is due to the dynamic changes in the lead-to-cord distance during patient’s movements, and the volume of neural tissue activated (VTA) by the stimulation.^4^ For instance, the spinal cord is closest to the lead when the patient is supine and farthest when prone. This dose inconsistency is further compounded by the cerebrospinal fluid (CSF) thickness changing along the cephalad-caudad neural axis.^4^^,^^5^ While the CSF thickness is lower in the cervical spine, the greater mobility of the neck (vs. lower back) makes therapy delivered more susceptible to inconsistent dosing.

With OL-SCS, variations in VTA with movement or postural shifts may cause changes to the therapy experience and efficacy. Smaller distances may be uncomfortable and larger lead-to-cord distances could result in sub-therapeutic dose.^6^ In many instances patients may adjust their setting for efficacy and/or to avoid uncomfortable stimulation but some of them may not revert back to the programmed amplitude.^7^ In a recent study comparing OL to CL, paresthesia-based SCS, subjects receiving OL therapy were found to have adjusted their therapy to below the programmed amplitude range and were underdosing (median) 49.3% of the time.^8^ Over time these patients may perceive under-dosed therapy as being ineffective, stop using the therapy and/or request an explant. A recent survey of patients receiving modern SCS waveforms found that although 83% are satisfied with overall pain reduction outcomes, over 70% proactively adjust stimulation to avoid therapy side effects.^7^ In total, 85% of the participants reported avoiding one or more activities of daily living due to a fear of under- or overstimulation and/or turning therapy off. With newer waveforms that typically employ higher frequencies (≥200 to 10 000 Hz), the over-dosing effects may present differently than uncomfortable paresthesia typically associated with lower rate stimulation and may include muscle cramping, nausea, tinnitus, or headaches.^9^

There are currently 2 solutions available to address the inconsistency of dosing due to changes in lead-to-cord distance with movement: One based on a posture sensor^6^ and another that uses biosignals.^10^^,^^11^ The posture sensor method relies on an accelerometer inside the neurostimulator that can detect gross changes in position such as from standing to supine and switches to pre-programmed, posture-specific amplitude settings. The biosignal method relies on detection of evoked compound action potentials (ECAPs). An ECAP is the summed activity of nerve fibers synchronously activated by the SCS pulse and characteristics of the ECAP can be used to adjust the amplitude in an ECAP-based CL system. It has the advantage of being more sensitive to the patient’s movements than the posture sensor and being able to make more rapid adjustments. By automatically adjusting the stimulation, these systems reduce the variability in the dose reaching the spinal cord and the burden of making manual adjustments. The durable benefits of an ECAP-based, CL-SCS system compared to a low-rate, paresthesia-based therapy, was recently demonstrated in a chronic low-back and leg pain population.^10^

Here we report CL performance and pain reduction outcomes from the Closed-Loop SCS study. The study is evaluating a flexible, ECAP-based, CL-SCS system that delivers a variety of waveforms, including differential target multiplexed SCS, across a range of amplitudes (relative to perception).^11^ The primary objective of the study was to demonstrate reduction in overstimulation with CL- versus OL-SCS. Secondary measures included pain-related outcome measures. Together, the primary and secondary measures, evaluated at the 1- and 3-month visits, respectively, will illustrate the system’s ability to maintain effective therapy without treatment limiting adverse effects.

## Methods

### Study design

**Figure 1.**
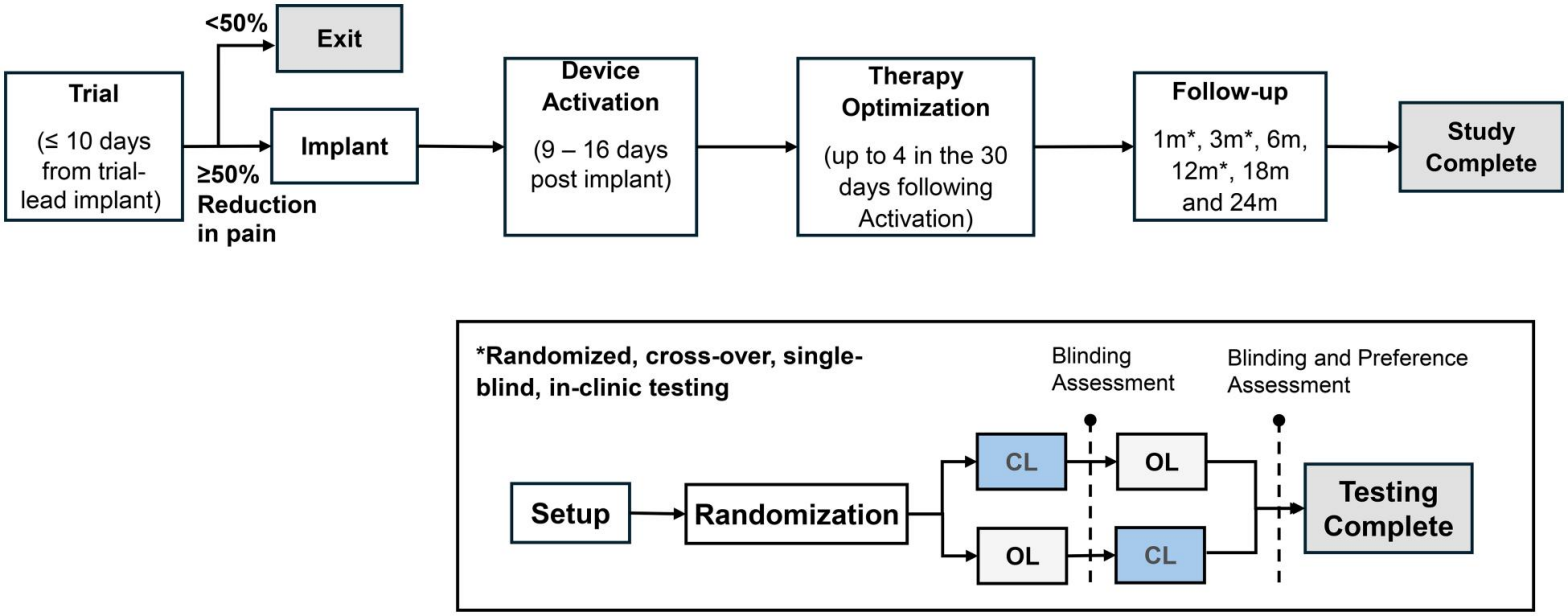
Study flow diagram. The study has 2 parts including a long-term follow-up phase out to 24 months from Device Activation and a randomized, crossover, in-clinic testing of CL performance. Subjects were blinded to the sequence of CL and OL periods for the duration of the in-clinic testing. At the end of the crossover testing, subjects were asked to state a preference for period 1 or 2. The randomization is only for the duration of the in-clinic testing and determines the sequence of CL and OL testing. At the end of the visit subjects left with their most used therapy setting active.

The Closed-Loop SCS study is a prospective, single-arm, multi-center (7 sites in Australia) trial with up to 24 months of follow-up post Device Activation (NCT05177354). The study includes in-clinic, randomized, blinded, crossover testing to assess the performance of the closed-loop feature at the 1-, 3-, and 12-month visits in the study design (see Figure 1, description below and in the Supplement). The longer-term follow-up (≤ 24 months) allows characterization of the durability of pain reduction outcomes and other benefits of the CL-SCS system. Follow-up visit timing is relative to Device Activation when the Inceptiv™ INS is programmed for therapy and occurs 9–16 days post implant. All serious adverse events (SAEs) and device, therapy or procedure related adverse events (AEs) were tracked from the time of study enrollment. CL performance and pain reduction outcomes through the 3-month visit are presented here.

The study is compliant with the principles outlined in the Declaration of Helsinki, ISO14155 and other applicable regulations. The study was approved by the local Ethics Committee prior to the start of enrollment. Subjects provided informed consent prior to participating in any study related activities and were compensated for their time. Key eligibility criteria include on-label indication for SCS implant, baseline pain VAS of ≥60 mm or more, stable dose of analgesics for at least 28 days prior to enrollment and absence of other pain conditions and refractory psychiatric comorbidities that could confound study outcomes. Study eligibility criteria are listed in Table S1. In addition to the eligibility criteria, subjects had to have ≥50% relief during SCS trial to be implanted with the study device.

The CL feature is intended to ensure consistent dosing, by adjusting therapy amplitude in proportion to the lead-to-cord distance, as determined from the ECAP signal (measured 50 times per second). Other studies have compared ECAP-based CL with OL, while applying low-rate tonic stimulation.^8^ This study included the use of CL with a mix of waveforms such as differential target multiplexed (DTM^TM^) SCS, that have demonstrated superior outcomes to low-rate tonic stimulation (with OL therapy).^12^^,^^13^

Subjects were programmed with therapy that included one or two waveforms (programs), at a comfortable amplitude relative to perception and discomfort threshold. Perception threshold (PT) is defined as the amplitude at which the sensation of stimulation disappears when decreasing stepwise. Discomfort threshold (DT) was defined as the amplitude at which patients could not tolerate the sensation of stimulation for more than a few minutes. The study did not incorporate a therapy comparison group as it is not intended to demonstrate the relative efficacy of CL compared to OL waveforms in reducing pain. SCS Therapy and CL programming are described in more detail in the Supplement and Figure S1.

Baseline assessments included pain visual analogue score (VAS), quality of life (PROMIS-29, EQ-5D-5L, SF-12), physical function (Oswestry disability index [ODI], Upper extremity function index [UEFI], neck disability index [NDI]), sleep quality (Pittsburgh sleep quality index (PSQI)), and profile of mood states (POMS). Subjects also identified a goal (eg, walk longer, household chores, self-care, etc) they wanted to achieve after SCS device implant. These assessments were repeated at the 3-, 6-, 12-, 18-, and 24-month visits per study design. Performance of the CL feature was tested in a controlled and repeatable manner during the 1-, 3-, and 12-month visits per study design (see Primary Objective section). CL performance and pain reduction outcomes through the 3-month visit are presented here. Additionally, subjects reported on their at-home experience with SCS therapy by responding to the following questions on a 5-point Likert scale (strongly disagree, disagree, neutral, agree, and strongly agree).

Question 1: I notice that the therapy is on.

Question 2: I’m afraid that if I move in certain ways my therapy will shock me or become too intense.

Question 3: I’m comfortable being physically active with my stimulation therapy turned on.

Question 4: I have to turn down or turn off my stimulation therapy in order to do certain activities.

### Statistics

#### Primary objective

The primary objective was to demonstrate that the proportion of subjects with low-back and/or leg pain having a reduction in overstimulation sensation with CL compared to OL, at the 1-month visit, exceeds a performance goal of 50%. Per the power analysis and sample size test of one proportion, 28 subjects are required to reject the null hypothesis, with 90% power, with a 1-sided type one error of 0.025. Hence, the first 28 subjects to complete the randomized, crossover testing at 1-month were included in the evaluation of the primary endpoint and constitute the Primary Analysis Set.

For this testing, subjects were randomized to receive OL followed by CL or vice versa. A visit-specific randomization listing was generated using a module within the Rave Electronic Data Capture (EDC) designed by the Sponsor’s Biostatistics team. Each randomization listing consisted of permuted blocks of 2 and 4 to maintain the balance of subjects receiving each randomization sequence. Subjects were blinded to the OL and CL setting for the duration of this testing.

For each testing period, that is, OL or CL, subjects performed 3–5 repetitions of one or more movements (eg, back arch, arm lift, leg raise, cough, etc) that simulates activities of daily living (ADL) and is known to result in overstimulation. For each repetition, subjects reported an intensity of sensation on a 5-point Likert scale from 0 to 4. Four indicates very strong overstimulation and zero none. Success was defined as average intensity during the CL testing being lower than during the OL period. A blinding assessment was performed at the end of each testing period. Additional details of this testing are provided in the Supplement and has been previously described in Will et al.^14^

#### Secondary objectives

The secondary objectives were to characterize pain relief by evaluating the efficacy responder rate. Responder rate is defined as the percentage of 3-month completers who experienced ≥50% reduction in overall pain VAS relative to baseline. The responder rate was also calculated for subjects with baseline low-back, leg and upper limb pain. A sensitivity analysis was performed on all implanted subjects with missing VAS scores at 3-months using multiple imputation.

This study does not include hypothesis testing for any of the secondary objectives. The sample size calculation was based on the precision estimate around the overall low-back/leg pain responder rate; precision is defined as one-half of the confidence interval (CI). Assuming a responder rate of 75% at the 3-month visit, two-sided alpha of 0.05 and exact binomial confidence interval, a sample size of 45 subjects would have a precision of 0.134 leading to a 95% CI of 0.60–0.87. Based on the attrition rates typically seen in SCS studies between Enrollment and the 3-month visit, the study design allowed for the enrollment of up to 140 subjects, to ensure ≥45 subjects completed the 3-month visit.

#### Additional measures

Unless otherwise stated, continuous variables are summarized with mean (standard deviation) or median (minimum, maximum or interquartile range) and categorical variables as numbers, percentages and 95% confidence interval.

## Results

### Subject disposition and baseline characteristics

**Figure 2.**
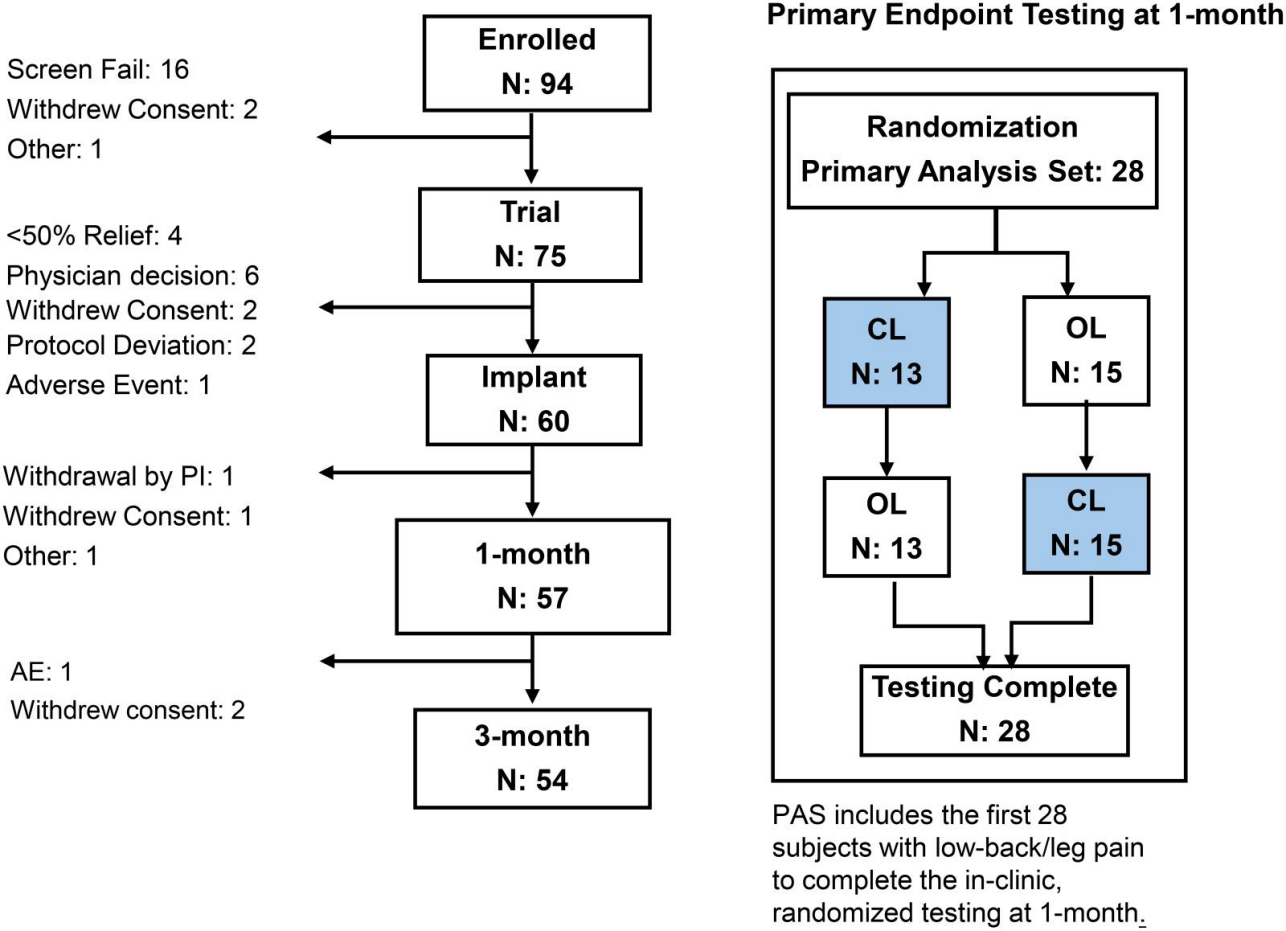
Subject disposition up to the 3-month visit and for the Primary Endpoint assessment. Counts at each visit indicate the number of subjects who completed the visit. The Primary Analysis Set (PAS) includes the first 28 subjects with Low back/leg pain and usable ECAPs to complete the 3-month visit. The 60 subjects implanted with the study device include 57 with low-back/leg pain and 3 with upper limb pain.

Study enrollment began in December 2021 and was completed in March 2023. A total of 94 subjects were enrolled in the study from 7 sites and 75 underwent a SCS trial. Of the 75 subjects that completed the SCS trial, 69 had ≥50% reduction in pain (92% trial period success rate) and 60/69 were implanted with the study device. CL performance and pain reduction outcomes through the 3-month visit are presented here. Subject disposition through the 3-month visit including reasons for early exits are shown in Figure 2.

In the 60 subjects implanted with the study device, the primary reason for chronic pain was persistent spinal pain syndrome type 2 occurring in 63% of subjects. Three subjects had upper limb pain and the rest low-back/leg pain. The baseline average Overall VAS was 80 mm, and the median (interquartile range [IQR]) time since onset of pain was 7 (3–12.5) years. A majority of the subjects in this study were in a severely disabled or worse state (per ODI) due to chronic pain and had multiple other major comorbidities at the time of enrollment. Baseline characteristics are summarized in Table 1.

**Table 1.** Baseline characteristics for all subjects implanted with the study device and the Primary Analysis Set.

Subject Characteristics	Implanted (*N* = 60)	Primary Analysis Set (*N* = 28)
**Age (years) – mean (SD)**	57.9 (14.1)	58.6 (13.5)
**Female—n (%)**	27 (45.0)	13 (46.4%)
**Primary indication—n (%)**		
PSPS—Type 2^19^	38 (63.3)	18 (64.3)
PSPS—Type 1^19^	17 (28.3)	9 (32.1)
CRPS—Type 1 and 2	5 (8.3)	1 (3.6)
**VAS (mm) – mean (SD)**		
Overall	80.3 (10.5)	79.5 (11.0)
Back	73.7 (19.9)	77.4 (15.7)
Leg	73.1 (22.7)	75.6 (17.8)
Upper limb	81.3 (6.4)	–
**Severely disabled or worse per ODI—n (%)**	49 (86.0)	24 (85.7)
**Years since onset of pain—median (Q1– Q3)**	7.0 (3–12.5)	7.0 (3.5–10)
**On opioids—n (%)**	45 (75.0)	21 (75.0)
**Number of analgesic medications—mean (SD)**	2.9 (1.9)	3.0 (1.9)
**Morphine milligram equivalents**		
Subjects with MME > 0 at Baseline—n (%)	42 (70.0)	19 (67.9)
MME—median (min–max)	75 (7.5–225)	75 (7.5–225)

Abbreviations: CRPS, complex regional pain syndrome; MME, morphine milligram equivalents PSPS, persistent spinal pain syndrome; Q1, first quartile; Q3, third quartile; SD, standard deviation.

Subjects with a primary indication of failed back syndrome, post-laminectomy pain, unsuccessful disk surgery, degenerative disk disease and radicular pain syndrome are grouped under the PSPS category. The absence of a history of spine surgery differentiates PSPS-T1 and PSPS-T2.

### Therapy and closed loop settings

**Figure 3.**
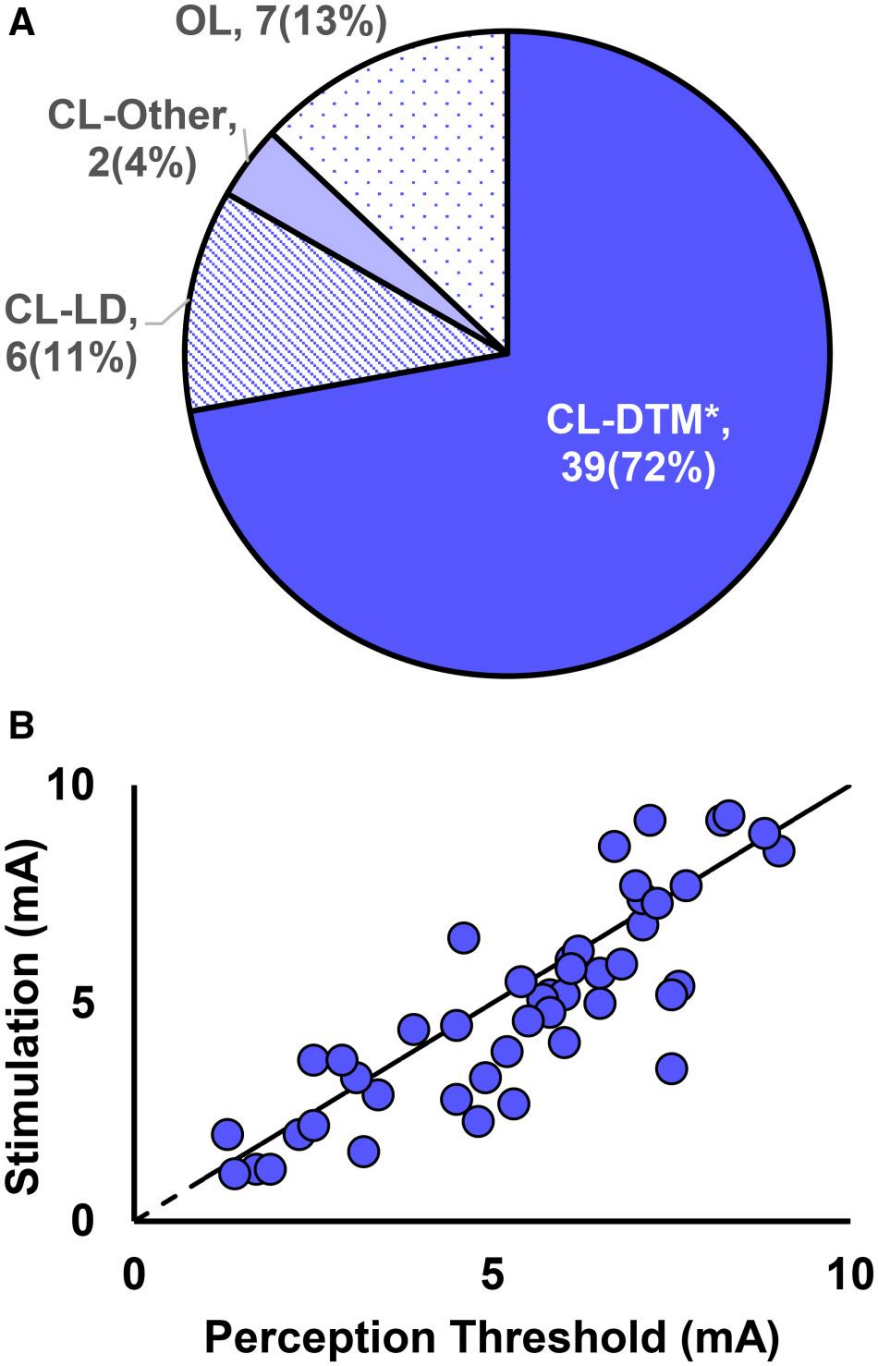
(A) Summary of the most used therapy for each patient over the 30 days preceding the 3-month visit. *DTM includes 1 low-rate (<200 Hz) and 1 high-rate (≥200–1200 Hz) program applied at 2 different targets. Of the 7 subjects with an OL waveform as their most used therapy group, 1 had a pacemaker that interfered with the CL feature, 4 others did not have consistent ECAPs at comfortable stimulation amplitudes, and 2 used a mix of OL-SCS and CL-SCS. (B) Perception threshold (PT) and Program1 amplitude for the most used therapy settings with the subject seated in a comfortable posture (ie, “rest” state). PT is defined as the amplitude at which the sensation of stimulation disappears when decreasing stepwise. PT is specific to the posture assumed by the person during programming. Stimulation amplitude was programmed to comfort near PT (ie, below, at or above perception). Program1 elicits the ECAP signal and provides therapy. When programmed to less than PT (below dashed line), ECAPs present during movement that cause the lead-to-cord distance to decrease. When stimulation amplitude is above perception, ECAPs are present at “rest.”

Eighty-seven percent of subjects had a closed-loop waveform as their most used therapy at the time of the 3-month visit. Of the 7 subjects that had OL-SCS as their most used therapy group, 4 did not have consistent ECAP signals at comfortable stimulation amplitudes, one subject had an implanted pacemaker that interfered with the CL feature and 2 others used a mix of OL-SCS and CL-SCS (Figure 3A).

In this study, subjects were programmed to comfort near PT. For the most used therapy group shown in Figure 3A, mean (SD) of stimulation amplitudes were 0.89 (0.27; *n* = 46) of PT for Program1 and 0.78 (0.47; *n* = 46) for Program2. Average DT was 1.3 (SD 0.2; *n* = 33) times PT for Program1. While both programs provide therapy, Program1 generates the ECAP signal to drive the CL algorithm. Figure 3B shows the stimulation amplitude and PT for Program1. This plot highlights the distribution of preference for below- (amplitude below the dashed line) and above-perception (amplitude above the dashed line) waveforms. Sixty percent of the subjects had below-perception waveforms, and the rest had above-perception waveforms as their most used therapy setting.

**Figure 4.**
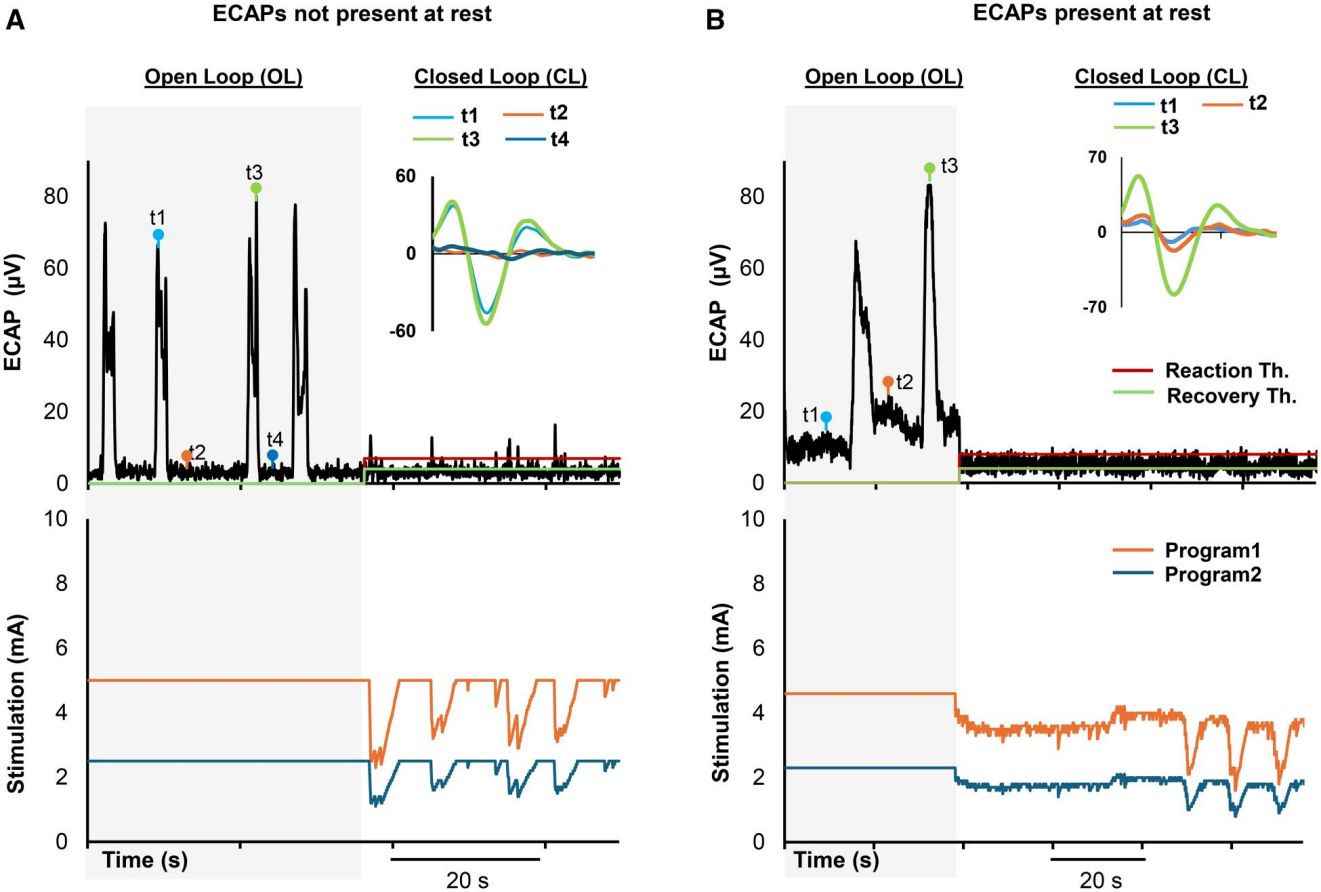
Examples of the CL feature adjusting stimulation with ECAPs present at rest or only during movement. ECAPs were measured in-clinic, at rest and during movement, with OL- and CL-SCS. The large variation in ECAP in the OL setting is due to the difference in the “dose” reaching the spinal cord and volume of neural tissue activated with changes in lead-to-cord distance as the subject performs a movement. With CL-SCS, amplitude was adjusted to provide a consistent dose, which is reflected in the significantly reduced ECAP variability relative to the OL period. Both examples are with DTM therapy with rates of 50 and 900 Hz for the 2 programs. (A) Example for CL engagement during movement when ECAPs present. Program1 and Program2 amplitudes were at 94% and 64% of perception threshold (PT), respectively. Program1 amplitude was below PT, there are no ECAPs at rest (eg, at t2 and t4). However, when the subject engages in movements that reduce the lead-to-cord distance, the VTA of in spinal cord increases, seen as a large change in ECAP amplitude (eg, at t1 and t3). (B) Example for CL engagement with ECAPs present at rest. Program1 and Program2 amplitudes were at 105% and 80% of PT, respectively. Program1 is above PT, ECAPs are present at rest (eg, at t1 and t2) and during movement (eg, at t3). The increase in ECAP size during movement can be seen in the example shown in the inset at t3.

Shown in Figure 4 are sample ECAP recordings that illustrate CL adjusting stimulation amplitude for a waveform programmed with below and above PT amplitude. The recordings were obtained during the 3-month, in-clinic visit as the subject engaged in movements that occur during ADLs. When the stimulation amplitude is programmed to below PT, ECAPs are measurable during activities that cause the lead-to-cord distance to decrease. Figure 4A shows CL responding by rapidly decreasing the amplitude when ECAP size exceeds the reaction threshold. Once the ECAP amplitude drops below recovery threshold the algorithm increases it back to the programmed setting. Figure 4B provides an example for stimulation amplitude programed above PT to provide a comfortable level of paresthesia coverage. In this case, CL maintains the preferred sensation by keeping the ECAP amplitude between the subject-specific thresholds. In both cases, CL ratiometrically adjusts the amplitude of Program2 using the ECAP signal generated by Program1. For example, if the amplitude of Program1 is reduced by 50%, Program2 will also be reduced by 50%.

### Primary endpoint

**Figure 5.**
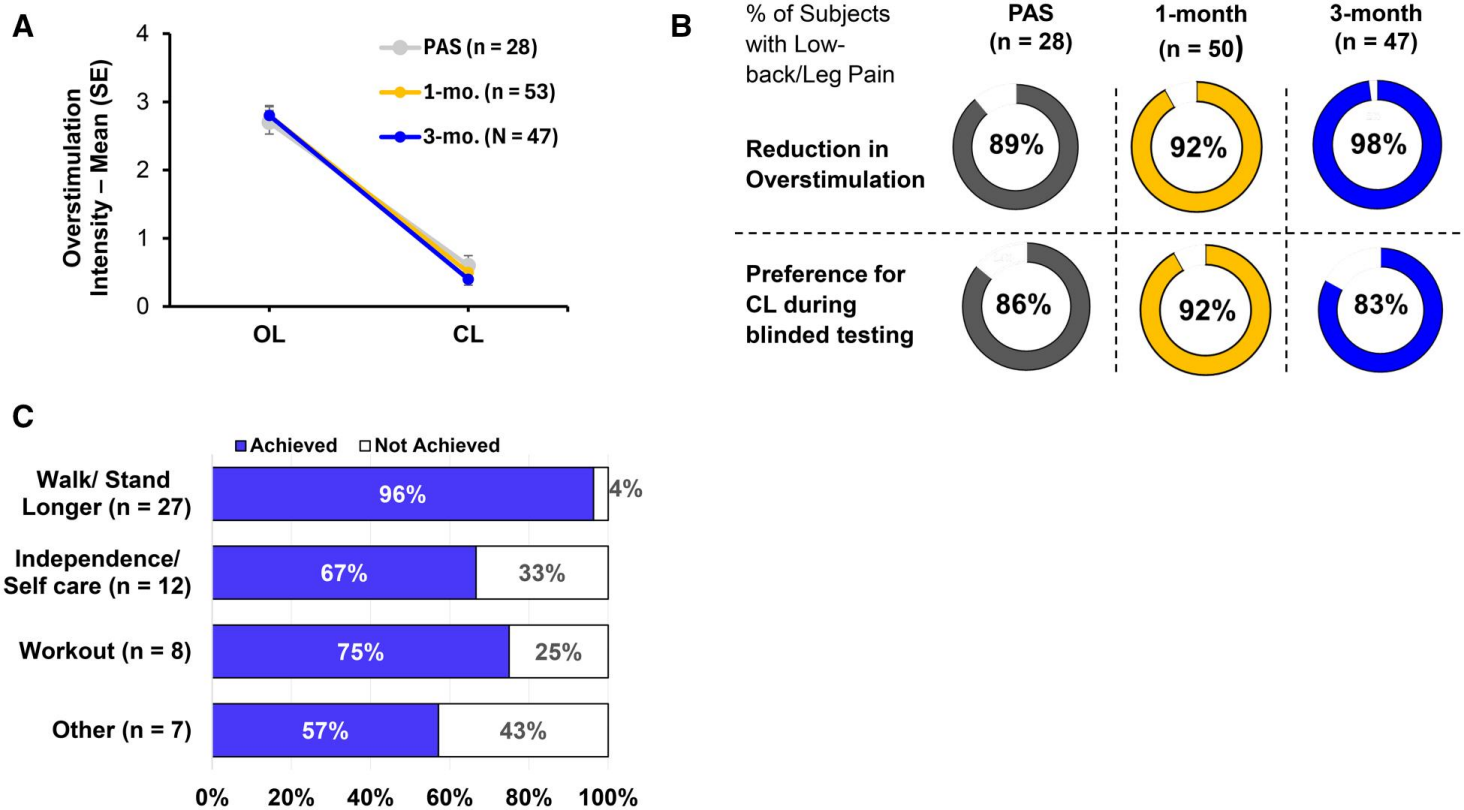
(A) Outcome of the randomized, crossover, in-clinic testing of CL feature performance at 1- and 3-months for subjects with low back/leg pain. Average intensity of overstimulation during protocol-specified activities with CL-SCS and OL-SCS; error bars show standard error from the mean. PAS—primary analysis set. All 3 subjects with upper limb pain also reported reduction in overstimulation at both time points. (B) Percent of subjects that experienced reduction in overstimulation with CL vs. OL and the proportion expressing a preference for the CL setting during blinded testing. For figures A and B, data from low-back/leg pain subjects that completed the 1- and 3-month visits are shown, to be consistent with the primary analysis set; 4 subjects did not have consistent ECAPs at comfortable stimulation amplitudes and did not complete the in-clinic testing at 1- and 3-month visits. (C) Proportion of subjects that were able to achieve the activity goal they identified at the Baseline visit by the time of the 3-month follow-up. Data for all 54 subjects, including 3 with upper limb pain that completed the 3-month visit are shown in the plot; those with upper limb pain had chosen self-care (*n* = 2) and return to work (*n* = 1) as their goal, and all 3 were successful.

The study successfully met its primary endpoint, demonstrating reduced overstimulation with CL during simulated ADLs during the testing performed at the 1-month in-clinic visit. The randomization testing was performed on all subjects with usable ECAPs (*n* = 54). The primary analysis set includes the first 28 subjects to complete the in-clinic, randomized crossover testing (see **Primary Objective** section for details on sample size). Twenty-five of the 28 randomized subjects included in the Primary Analysis Set had lower average overstimulation intensity with CL compared to the OL period (Figure 5A) leading to a success rate of 89.3% (25/28 subjects; 97.5% lower confidence bound: 71.8%; *P* < .001; binomial exact test). Three subjects were counted as failures for the primary endpoint assessment—two subjects reported equal intensity during both periods and the third experienced stronger overstimulation during the CL testing period; this last subject was found to have received the incorrect sequence of CL and OL testing relative to their assigned randomization group due to human error. Key baseline characteristics were similar between the two randomization groups (Table S2). No significant effect was observed for the sequence of CL/OL (ie, testing period; *F*-statistic: 0.01; *P* value: .91) or the interaction between period and CL/OL (F-statistic: 2.5; *P* value: .13), signifying no period or carryover effects. Overstimulation reduction and preference outcomes were consistent at the 3-month visit (Figure 5B). All 3 subjects with upper limb pain also reported a reduction in overstimulation with CL (vs OL) and a preference for the CL setting at 1 month and 3 months.

The reduction in therapy inconsistency during the CL testing period, and consequently the intensity of overstimulation, is reflected in the reduced ECAP amplitude variability (examples in Figure 4**)**. The coefficient of variation (SD/mean) of the ECAP amplitude (*n* = 40) at 3 months, was 61% during the CL period compared to 128% during the OL phase of testing.

### Real world performance of the Closed-Loop algorithm

During at home use, over 80% of subjects reported feeling comfortable engaging in ADLs. The 20% (*n* = 11) that reported not feeling comfortable engaging in ADLs did not report fear of therapy side effects or feel the need to manually adjust therapy settings which suggests they may have had other reasons for activity avoidance (eg, muscle deconditioning). Ninety-one percent of the subjects were able to complete their activity goals identified during baseline assessments. In general, activity goals centered around becoming more self-sufficient, including being able to stand/walk longer, and care for self and/or family (Figure 5C).

### Improvement in pain, quality-of-life and physical function at 3-months

**Figure 6.**
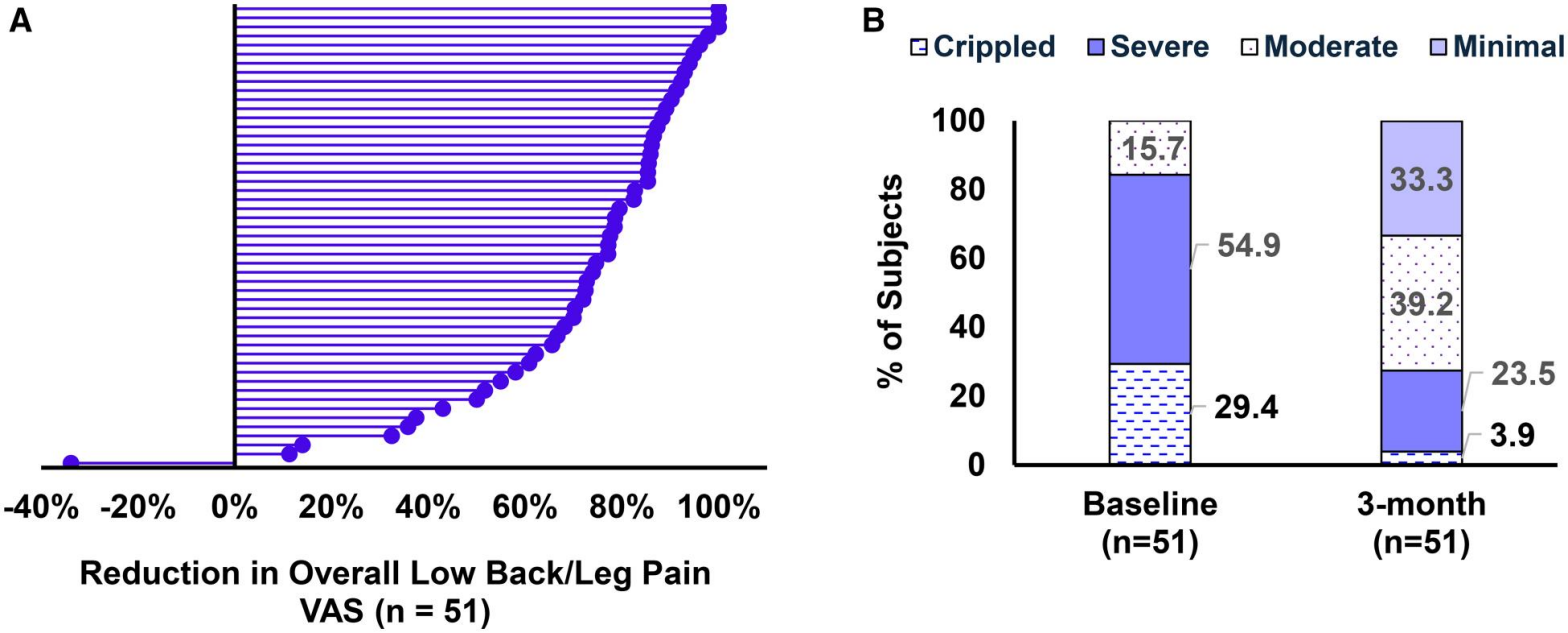
(A) Reduction in overall low-back/leg pain visual analogue score at 3 months post device activation relative to Baseline. All three subjects with upper limb pain had ≥50% reduction in pain. (B) Improvement in physical function as measured by the Oswestry disability index for subjects with low-back/leg pain. Per the ODI instrument, individuals are categorized as having minimal, moderate, or severe disability when scores are between 0–20%, 21–40%, and 41–60%.

Forty-nine of the 54 subjects that completed the 3-month visit reported ≥50% reduction in overall pain resulting in a responder rate of 86%.;82% reported improvement in disability as measured by ODI (Figure 6A and 6B). Baseline and 3-month VAS, percent change and subjects with ≥ 50% reduction in VAS is summarized in Table 2. Subjects reported clinically meaningful improvements in quality-of-life and physical function (Table 3 and Table S3). Although this study did not include an opioid weaning protocol, 35% of subjects that completed the 3-month visit reported reducing/stopping the use of these analgesics. Median MME at 3 months was 40 (min-max: 0–365) vs 75 (min-max: 7.5–225) at the time of enrollment. One subject had increased use to due to an AE that occurred 30 days before the 3-month visit.

**Table 2.** Reduction in pain visual analogue score at 3 months post device activation.

VAS (mm)	Baseline	3-month	% Change Mean (SD)	Responder Rate (95% CI)
Overall (n = 51)	78.5 (10.0)	22.2 (19.7)	71.5 (25.9)	86.3 (73.7–94.3) %
Low-back (n = 43)	78.3 (9.5)	19.3 (18.0)	75.4 (23.3)	88.4 (74.9–96.1) %
Leg (n = 41)	79.2 (11.3)	26.3 (25.9)	67.2 (32.5)	73.2 (57.1–85.8) %
Upper limb (n = 3)	84.3 (2.3)	17.3 (16.2)	79.3 (19.6)	100%

Abbreviations: CI, confidence interval; SD, standard deviation; VAS, visual analogue score.

**Table 3.** Improvements in physical function, quality of life, and satisfaction at 3-months from Device Activation.

Patient Reported Outcomes	Baseline	3-month	Change
**EQ 5D 5 L QoL (*n* = 54)** ^20^			
Index Score—mean (SD)	0.35 (0.23)	0.74 (0.18)	0.39 (0.23)
Index score change ≥ MCID of 0.074—n (%)^21^	–	–	49 (90.7)
Improved health state—*n* (%)	–	–	40 (74.1)
**Patient-Reported Outcomes Measurement Information (PROMIS-29; *n* = 54)**			
5-point change in ≥ 3 domains—*n* (%)	–	–	43 (79.6)
**Overall Short Form 12 QoL Questionnaire (SF-12; *n* = 54)** ^22^			
PCS—mean (SD)	26.3 (5.9)	35.5 (10.5)	9.2 (9.4)
Change ≥ MCID of 6—*n* (%)	–	–	33 (61.1)
MCS—mean (SD)	41.9 (11.6)	48.3 (11.5)	6.4 (14.2)
Change ≥ MCID of 7—*n* (%)	–	–	28 (51.9)
**Oswestry Disability Index (ODI; *n* = 51 with Low-back/Leg Pain)**			
ODI Score (%) – mean (SD)	53.8 (13.2)	30.0 (17.2)	−23.9 (16.9)
≥10 percent reduction—*n* (%)^23^	–	–	44 (86.3)
Improvement in Disability—*n* (%)	–	–	42 (82.4)
**Upper Extremity Functional Index (UEFI; *n* = 3 with Upper limb pain)**			
UEFI—mean (SD)	42.2 (11.1)	48.9 (2.9)	6.7 (12.5)
**Neck disability index (NDI; *n* = 3 with Upper limb pain)**			
NDI Score—mean (SD)	63.3 (4.2)	38.7 (7.0)	−24.7 (10.3)
Change ≥ MCID of 15—*n* (%)^24^	–	–	3 (100)
**Profile of Mood States (POMS; *N* = 53)**			
TMD-T Score—mean (SD)	63.4 (14.7)	52.6 (12.3)	−10.8 (16.6)
Change ≥ MCID of 10—*n* (%)^25^	–	–	27 (50.9)
**Pittsburgh Sleep Quality Index (PSQI; *n* = 52)**			
PSQI—mean (SD)	13 (4.5)	8.8 (4.0)	−4.2 (4.9)
Change ≥ MCID of 3—*n* (%)^26^	–	–	32 (61.5)
**Patient Global Impression of Change to Limitations, Symptoms, Emotions and Overall QoL (*n* = 54)**			
Better or a great deal better—*n* (%)	–	45 (83.3)	–
**Satisfaction with Therapy (*n* = 54)**			
Very satisfied/satisfied—*n* (%)	–	54 (100)	–
Definitely/Probably recommend this therapy—*n* (%)	–	53 (98.1)	–

Abbreviations: EQ5D, Euro QoL 5-dimension 5-level assessment; MCID, minimum clinically important difference; MCS, mental component summary; QoL, quality of life; PCS, physical component summary; SD, standard deviation; TMD, total mood distribution.

### Safety

There were no unanticipated adverse events in this study. Adverse events are summarized in Table 4. The rate of ADEs and SADEs is similar to previous SCS studies. A total of 16 serious adverse events were reported by 14 subjects that were not related to device, therapy or procedure. The SAEs observed in this study highlight the presence of other major co-morbidities in the study cohort including cardiovascular, metabolic and neurological conditions.

**Table 4.** Summary of adverse events.

	Events (Subjects, % of Subjects)		
Event Type	Pre-INS Implant (*N* = 75)	Post-INS Implant (*N* = 60)	Total (*N* = 75)
All adverse device events^a^	4 (4, 5.3%)	19 (13, 21.7%)	23 (17, 22.7%)
Serious adverse device events^a^	3 (3, 4.0%)	4 (4, 6.7%)	7 (7, 9.3%)

a Device, Therapy or Procedure Related adverse event as determined by Investigator.

The 7 SADEs include infection (IPG pocket = 1; lead entry site = 1), 1 each of IPG pocket hematoma, post dural puncture headache, atrial fibrillation, allergic reaction to antibiotics, and pain at IPG site.

## Discussion

This study demonstrates the feasibility of utilizing ECAP-based, CL-SCS with a mix of tonic and multiplexed waveforms programmed near (below, at, or slightly above) perception in permanently implanted subjects. The study was intentionally designed to allow for the evaluation of the CL in a representative sample of chronic pain patients typically implanted with SCS devices. The lack of a control group and open-label nature of the study measuring pain-related outcomes limits the ability to determine the magnitude of placebo effects. However, continued follow-up over the course of the study, to 24 months, should reveal the impact of any placebo effects that are unlikely to persist for 2 years.

In evaluating the performance of CL, the study successfully met its primary endpoint with 89% of subjects reporting reduction in overstimulation during in-clinic testing at 1-month (Figure 5). The in-clinic, CL performance data are complemented by the at-home experience of the subjects; over 80% reported feeling comfortable engaging in ADLs without fear of therapy side effects or having to turn therapy down or off prior to activities. In a recent survey of OL- SCS device users, 70% of participants reported proactively adjusting their therapy settings up/down/off to avoid potential side effects of dose inconsistency.^7^ An outcome that is possibly most important to the subjects in this study, 82% of participants were able to achieve the activity goal they had identified at the Baseline visit. The achievement of goals is likely due to both reduction in pain and consistency of therapy dosing (or reduced side effects) with CL. These outcomes contrast with the survey of patients using OL-SCS therapy, where 85% of participants report avoiding one or more activities to avoid under- or overstimulation including laughing and coughing.^7^ Of the 10 subjects that were unable to achieve their activity goal in this study, only one described the sensation of stimulation as unpleasant. This suggests that the activity limitation at 3 months may be due to the prolonged duration of disability (as reflected by the baseline ODI or UEFI-15 score) and consequent muscle deconditioning secondary to the onset of the painful condition.

As demonstrated in this study, 1 benefit of this flexible closed loop approach is that subjects can be programmed above or below perception threshold (PT) depending on their preference. Given that ECAPs typically emerge near or above PT,^15^^,^^16^ 1 of 2 programming approaches can be used depending on the presence of ECAPs at rest. If there is an ECAP present, the CL feature reduces the ECAP variability by increasing or decreasing the stimulation amplitude when the ECAP exceeds subject-specific thresholds (Figure 4B). By improving the consistency of dosing, CL maintains stimulation above the level required for therapy efficacy and below any treatment-limiting overstimulation. This approach is particularly well suited to paresthesia-focused therapy (Figure 4B) where stimulation amplitude is nearly always above PT and is similar to previously published ECAPs-based, CL-SCS programming approaches.^8^

It is important to consider patient comfort when programming the stimulation amplitude given that on average DT was 1.3xPT at the time of the 3-month visit similar to what has been previously reported by several other studies.^15^^,^^17^^,^^18^ Given that many SCS patients prefer to not feel the sensation of stimulation continuously, the approach shown in Figure 4A may be used to adjust therapy amplitudes when programmed below ECAP threshold and thus below or near PT. Since PT varies with postural shifts, the ECAP will be measurable when the patient assumes a posture that reduces the lead-to-cord distance, and the CL feature will respond by adjusting the stimulation amplitude to keep the ECAP between the subject-specific thresholds.

While 87% of subjects in this study were programmed with CL-SCS, a small number of subjects (13%) used OL therapy. Of these 7 subjects, 1 person had ECAPs, but there was interference from another active medical device with the functioning of the CL feature. Future studies should further investigate the impact of additional active medical devices such as pacemakers on sensing performance of CL SCS devices, evaluating across factors including stimulation parameters of the pacemaker, the orientation of the SCS lead relative to the pacing lead, characteristics of the SCS lead-tissue interface, and any other biophysical factors (such as CSF thickness) that affect coupling of the electrical field between devices. Four others did not have consistent ECAPs at comfortable stimulation amplitudes and the others used a mix of OL and CL therapy.

A variety of reasons could underly why ECAPs may be difficult to detect using some electrode configurations in some patients. First, greater CSF thickness could negatively impact the ability to record ECAPs because a large amount of charge injection would be needed to activate the spinal cord near the stimulation target and the CSF would also effectively reduce the ECAP signal near the recording area. Other patient specific factors could include the presence of scar tissue that could have a similar effect.

Notably, the CL feature is not intended to be a therapy waveform but a tool that automatically adjusts the stimulation amplitude to account for the changing lead-to-cord distance with patient movements thereby enabling consistency of dosing. Thus, the same mechanism of action for analgesia is likely engaged for CL waveforms as the OL counterparts. Improvement in pain, quality-of-life and physical function were reported by over 80% of subjects and the outcomes are similar to previous studies that evaluated DTM delivered without CL.^12^^,^^13^ While the absence of ECAPs does not limit the device from delivering SCS therapy, ECAPs when present could be used to provide the patient with a more consistent therapy experience.

## Conclusions

The evidence presented here supports the performance of an ECAP-based, CL-SCS system with a novel approach to address inconsistent dosing resulting from the dynamically varying lead-to-cord distance. The CL feature continuously senses ECAPs (50 times per second) and reacts when needed by reducing, increasing or holding amplitude. This enables the use of the CL feature with both below and above perception stimulation amplitudes, without requiring a continuous presence of ECAP signals. We also demonstrate the feasibility of multiplexing additional waveforms to allow patients access to newer therapy such as DTM with the added benefits of CL. Specifically, improved dose consistency can be achieved by ratiometrically adjusting the amplitude of the multiplexed waveforms using the ECAP signal generated by Program1. Additional ongoing follow-ups of up to 24 months will evaluate the long-term benefits of this unique approach both in terms of health-related quality-of-life and experience with therapy.

## Supplementary Material

pnaf058_Supplementary_Data
