# Supplementary material for: Early outcomes with a flexible ECAP based closed loop using multiplexed spinal cord stimulation waveforms—single-arm study with in-clinic randomized crossover testing
Source: Pain Med. 2025 May 16;26(11):773–82. doi: 10.1093/pm/pnaf058 (PMC12585107; doi:10.1093/pm/pnaf058)
Supplement: pnaf058_Supplementary_Data [file pnaf058_supplementary_data.docx]

**Early Outcomes with a Flexible ECAP Based Closed Loop Using Multiplexed Spinal Cord Stimulation Waveforms – Single-arm Study with In-clinic Randomized Crossover Testing**

Vahid Mohabbati, MD,^1^ Richard Sullivan, MBChB,^2^ James Yu, MD,^3^ Peter Georgius, MBBS,^4^ Charles D Brooker MBChB,^5^ Malgorzata Siorek, PhD,^6^ Nancy L McClelland, BSN, ^6^ Filippo Coletti, MS, ^7^ Xiaoxi Sun, MA, ^6^ Abi Franke, PhD, EMBA, ^6^ Marc A Russo, MBBS^8^

**Supplemental Table 1.** Study Eligibility Criteria

| Type | Criteria |
| --- | --- |
| Inclusion | 1. 18 years of age or older.  2. Candidate is undergoing Medtronic SCS device trial for chronic, intractable pain of the trunk and/or limbs due to Failed Back Surgery Syndrome (FBSS), Complex Regional Pain Syndrome (CRPS), or other chronic neuropathic pain without history of  surgical interventions.  **Note:** The sponsor recommends not enrolling patients with chronic pain due to conditions that do not have adequate evidence to support the use of SCS (e.g., post-herpetic neuralgia, focal CRPS etc.)  3. If being treated for low-back and/or leg pain,   - the baseline overall^ VAS is ≥ 60 mm and - baseline back and/or leg pain VAS is ≥60 mm.   ^average overall pain in the back and/or leg in the 72 hours prior to the baseline visit, measured using VAS.  4. If being treated for upper limb pain, baseline VAS is ≥ 60 mm for upper limb pain.  5. On stable (no change in dose, route, or frequency) prescribed pain medications being used for back and/or leg pain or upper limb pain, as determined by the investigator, for at least 28 days prior to device trial.  6. Willing and able to provide signed and dated informed consent.  7. Willing and able to comply with all study procedures and visits.  **Note:** The sponsor strongly recommends only enrolling patients capable of comprehending and consenting in English. |
| Exclusion | 1. Indicated for an SCS device to treat stable intractable Angina Pectoris, Peripheral Vascular Disease of Fontaine Stage III or higher, or Diabetic Peripheral Neuropathy  2. Previously trialed or implanted with spinal cord stimulator, peripheral or vagus nerve stimulator, deep brain stimulator or an implantable intrathecal drug delivery system.  3. Currently participating, or plans to participate, in another investigational study unless written approval is provided by the Medtronic study team.  4. Major psychiatric comorbidity or other progressive diseases that may confound study results, as determined by the Investigator.  5. Serious drug-related behavioral issues (e.g., alcohol dependency, illegal substance abuse), as determined by the Investigator.  **Note:** The Sponsor recommends excluding patients on ≥100 MME of opioids/day.  6. Pregnant or planning on becoming pregnant (if female and sexually active, subject must be using a reliable form of birth control, be surgically sterile, or be at least 2 years post-menopausal)  7. Be involved in an injury claim or under current litigation.  **Note:** this includes patients that are the beneficiary of a successful injury claim. |

## Study Device and Programming

Subjects were implanted with two eight-contact cylindrical leads (Vectris trial lead, Model 977D2; Vectris SureScan MRI, Model 977A2; Medtronic Inc.); none of the subjects were implanted with surgical leads through the 3-month visit. For those with low-back/leg pain, the leads were typically placed to cover T8-T11 and for those with upper limb pain the C2-C5 region, per the implanter’s standard practice. For the trial, a single-use, wireless, battery powered, external neurostimulator was used to provide therapy (Wireless external neurostimulator, Model 97725; Medtronic Inc.); the duration of the trial was no more than 10 days from the implant of the trial lead. The Inceptiv™ neurostimulator (Model 977119; Medtronic Inc.), with the ECAP-based closed-loop feature, was implanted for the permanent system.

Subjects were typically programmed with multiplexed waveforms. For those programmed with DTM, one low-rate program at 50 Hz was multiplexed with a high-rate program in the frequency range of 200 – 1200 Hz and pulse widths between 170 - 200 µs. Other multiplexed waveforms may have included a second program with frequencies between 50-1200 Hz and pulse widths between 60 – 1000 µs. All stimulation parameters, including the waveform type, could be adjusted by the clinician as necessary based on the subject’s unique pain pattern. Stimulation amplitude for both programs was set as a percent of perception threshold to provide a comfortable experience to the subject.

Once the therapy settings were configured, the closed-loop feature was enabled to ensure consistency of dosing. (1) The programming of CL features, including setting maximum target amplitude and subject-specific ECAP thresholds is summarized in **Supplemental Figure 1**. When using the CL feature, Program1 is at 50 Hz and generates the ECAP and Program2 allows for interleaving additional therapy waveforms. The algorithm uses ECAPs sensed from the same lead that is delivering Program1 to adjust the amplitude of both programs ratiometrically; for example, a 50% change to Program1 would correspond to a 50% change in Program2. The algorithm uses two subject-specific thresholds – the reaction and recovery thresholds to determine when adjustments need to be made to the stimulation amplitude (**Figure 4**). In addition, the stimulation amplitude is limited to not go above the target amplitude set in clinic for safety.

Therapy settings and the CL feature were programmed at the Device Activation visit (9 – 16 days post device implant). The interval between device implant and activation is particularly relevant for ECAP-based, CL-SCS owing to the well-documented changes that occur at the electrode-tissue interface leading to encapsulation of the lead in the perioperative period. Accordingly, an allowance for stabilization of this interface may limit opportunity for inappropriate changes to stimulation parameters. The inflammatory response at the lead implant site leads to significant changes in impedance in the first 4-10 days and begins to stabilize thereafter; these changes affect the shape of the applied electric field, its nerve tissue recruitment properties and the signal-to-noise ratio of ECAPs.(2,3) In addition to these changes, recent work in the rodent spared nerve injury model of pain found that ECAP latency and thresholds vary significantly in the first 7 days post-implant and stabilize thereafter.(4) After the Device Activation visit, therapy settings were further optimized to achieve ≥50% pain reduction during scheduled visits (up to 4) prior to the 1-month follow-up and if necessary, the CL feature was also adjusted.


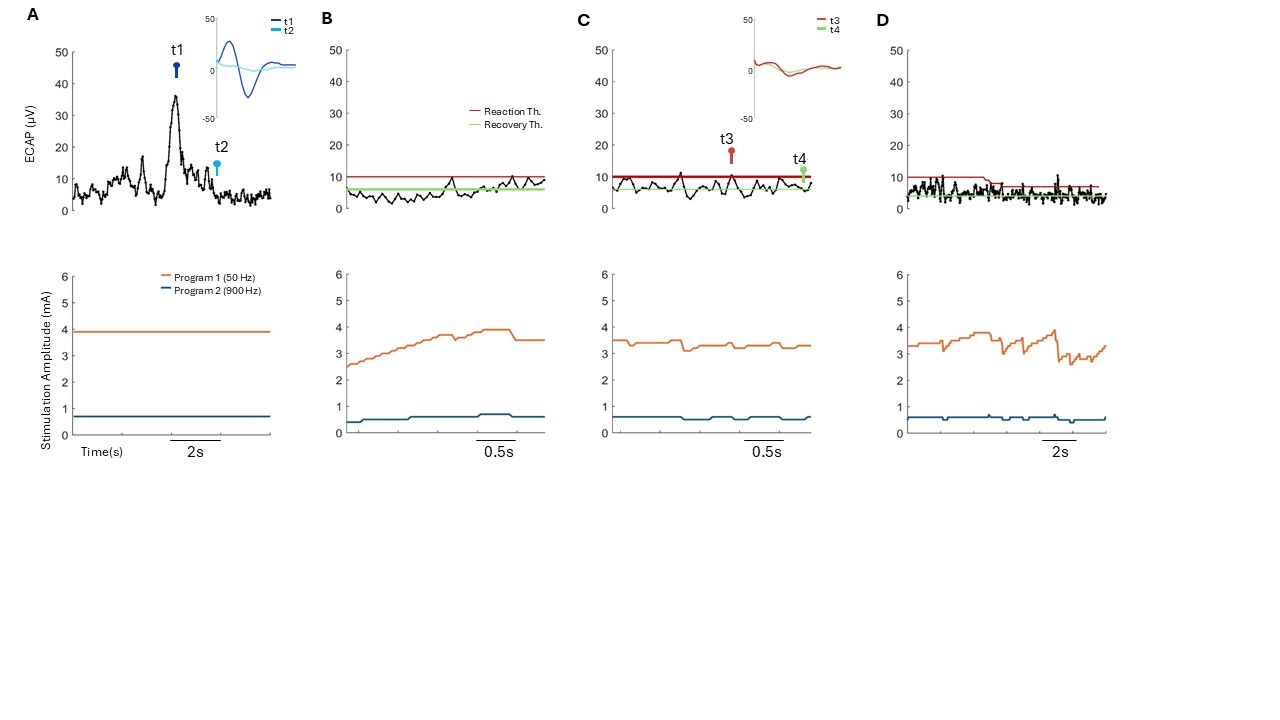


**Supplemental Figure 1**: Steps summarizing the programming of CL-SCS therapy. Programming starts with setting up the target amplitudes for both Programs based on operator experience and subject feedback. Once the target amplitudes were set, ECAPs were evaluated in the Open Loop condition with the subject at rest and while performing movements **(A)**. Next, the subject-specific thresholds were set, such that ECAP amplitudes below the Recovery Threshold cause an increase in stimulation amplitude **(B)** and ECAPs above the Reaction Threshold cause a decrease in stimulation amplitude **(C)**. During CL adjustment, stimulation will never exceed the target amplitude value for either Program1 or Program2. These thresholds were further refined for comfort by asking the subject to perform movements and observing the CL adjusting stimulation **(D)**.

## In-clinic CL Performance Testing

The in-clinic component of the study used the perceived intensity of stimulation as a metric of CL performance. This study design aimed to demonstrate CL performance with repeatable movements that occur naturally throughout the day and are known to cause transient increase/decrease in VTA by stimulation. For the duration of in-clinic testing, subjects were randomized in a cross-over design to either receive CL testing followed by OL or vice versa. During each in-clinic testing phase, subjects were asked to perform 3-5 repetitions of one or more of the activities (from the list below) and verbally rate the intensity of stimulation sensation. These movements were chosen to simulate actions that occur routinely during activities of daily living (e.g., reaching for things on a shelf, stretching, etc.). For each subject, one or more movements were identified from the list below that consistently resulted in overstimulation sensation.

- Back arch: the subject arches their back, as if to stretch.
- Arm raise: the subject raises both of their arms above their head, as if to reach something on a shelf.
- Leg lift: from a standing position, subject lifts either the right or left leg, as if walking up stairs. Alternatively, the subject could lift both legs from a seated posture.
- Head turn: turning the head from a seated or standing position as if to look at someone/something behind the person.
- Leaning forward from a seated or standing position
- Cough: subject coughs strongly
- Laugh: subject simulates laughter
- Torso twist: from a seated/standing position, subject twists their torso, as if to pick up something.
- Valsalva maneuver: similar to ‘bearing down’ while voiding.

Subjects were blinded to the CL or OL setting for the duration of the in-clinic testing. After each testing period, a blinding assessment was performed. At the end of the cross-over testing, subjects were asked to state their preference for the first or second testing period (i.e., CL or OL). Subjects self-rated the intensity of overstimulation on a 5-point Likert scale for each repetition of movement (no, mild, moderate, strong or very strong). Responses were converted to numerical values (0=no overstimulation, 4 = very strong overstimulation) and averaged across all activities performed in that testing period. Successful reduction in overstimulation was defined as average intensity during the CL period being lower than the OL period; if the average for both testing periods were the same, it was considered a failure for the primary endpoint evaluation. Given the crossover design of the primary objective, period or carryover effects on the average overstimulation intensity was tested using a linear mixed effects model. A pre-determined washout period was not included between the CL and OL testing periods because these overstimulation sensations were a posture dependent transient event that reduced in intensity or stopped completely when the subject returned to the relaxed, seated, posture. This design has been previously described in Will et al. (1)

**Supplemental Table 2.** Baseline Characteristics by Randomization Sequence for the Primary Analysis Set

| **Characteristic** | **OL to CL**  **(n = 15)** | **CL to OL**  **(n = 13)** | **p-value** |
| --- | --- | --- | --- |
| Age – mean (SD) years | 59.8 (13.4) | 57.2 (14.1) | 0.625^a^ |
| Female – n (%) | 8 (53.3%) | 5 (38.5%) | 0.476^b^ |
| Primary Indication – n (%) |  |  |  |
| PSPS – Type 2 | 7 (46.7%) | 11 (84.6%) |  |
| PSPS – Type 1 | 8 (53.3%) | 1 (7.7%) | 0.016^b^ |
| CRPS – Type 1 and 2 | 0 (0 %) | 1 (7.7%) |  |
| Abbreviation: SD – standard deviation; OL – open loop; CL – closed loop; PSPS – persistent spinal pain syndrome; CRPS – complex regional pain syndrome.  ^a^ t-test  ^b^ Fisher’s exact test | | | |

**Supplemental Table 3**: Absolute and percent change in patient reported outcomes.

| Patient Reported Outcomes | Baseline | 3-month | Change |
| --- | --- | --- | --- |
| **EQ 5D 5L QoL (n = 54)** | | | |
| Index Score – Mean (SD) | 0.35 (0.23) | 0.74 (0.18) | 0.39 (0.23) |
| % Change – Median (Q1 – Q3) | – | – | 115.1 (40.0, 349.0) |
| **Patient-Reported Outcomes Measurement Information (PROMIS-29; n = 54)** | | | |
| Ability to Participate in Social Roles/Activities T-Score – Mean (SD) | 37.0 (7.4) | 44.3 (8.3) | 7.2 (8.0) |
| % Change – Median (Q1 – Q3) | – | – | 17.1 (7.2, 39.2) |
| Physical Function T-Score – Mean (SD) | 34.4 (6.1) | 40.1 (7.0) | 5.7 (6.2) |
| % Change – Median (Q1 – Q3) | – | – | 14.6 (6.1, 25.1) |
| Anxiety/Fear T-Score – Mean (SD) | 56.7 (10.4) | 51.7 (10.3) | -5 (11.2) |
| % Change – Median (Q1 – Q3) | – | – | -7.8 (-17.4, 0.0) |
| Sleep Disturbance T-Score – Mean (SD) | 60.2 (9.5) | 52.0(8.4) | -8.2 (8.8) |
| % Change – Median (Q1 – Q3) | – | – | -14.9 (-20.7, -3.8) |
| Pain Interference T-Score – Mean (SD) | 69.0 (5.9) | 58.5 (7.8) | -10.5 (7.3) |
| % Change – Median (Q1 – Q3) | – | – | -13.5 (-21.6, -7.2) |
| Fatigue T-Score – Mean (SD) | 59.3 (10.4) | 51.3 (9.0) | -8.0 (10.1) |
| % Change – Median (Q1 – Q3) | – | – | -11.9 (-23.8, 0.0) |
| Depression/Sadness T-Score – Mean (SD) | 55.8 (10.6) | 52.4 (10.4) | -3.4 (12.4) |
| % Change – Median (Q1 – Q3) | – | – | -6 (-19.9, 0.5) |
| **Overall Short Form 12 QoL Questionnaire (SF-12; n = 54)^27^** | | | |
| PCS – Mean (SD) | 26.3 (5.9) | 35.5 (10.5) | 9.2 (9.4) |
| % Change – Median (Q1 – Q3) | – | – | 37.4 (13.6, 58.8) |
| MCS – Mean (SD) | 41.9 (11.6) | 48.3 (11.5) | 6.4 (14.2) |
| % Change – Median (Q1 – Q3) | – | – | 15.1 (-6.3, 46.4) |
| **Oswestry Disability Index (ODI; n = 51 with Low-back/Leg Pain)** | | | |
| ODI Score (%) – Mean (SD) | 53.8 (13.2) | 30.0 (17.2) | -23.9 (16.9) |
| % Change – Median (Q1 – Q3) | – | – | –44.4 (-64.5, -31.8) |
| **Upper Extremity Functional Index (UEFI; n = 3 with Upper limb pain)** | | | |
| UEFI – Mean (SD) | 42.2 (11.1) | 48.9 (2.9) | 6.7 (12.5) |
| **Neck disability index (NDI; n = 3 with Upper limb pain)** | | | |
| NDI Score – Mean (SD) | 63.3 (4.2) | 38.7 (7.0) | -24.7 (10.3) |
| **Profile of Mood States (POMS; N = 53)** | | | |
| TMD-T Score – Mean (SD) | 63.4 (14.7) | 52.6 (12.3) | -10.8 (16.6) |
| % Change – Median (Q1 – Q3) | – | – | -15.9 (-30, -2.2) |
| **Pittsburgh Sleep Quality Index (PSQI; n = 52)** | | | |
| PSQI – Mean (SD) | 13 (4.5) | 8.8 (4.0) | -4.2 (4.9) |
| % Change – Median (Q1 – Q3) | – | – | –32.5 (-50, -4.7) |
| **Abbreviations:** EQ5D – Euro QoL 5-dimension 5-level assessment; MCID – minimum clinically important difference; QoL – quality of life; SD – standard deviation; PCS – physical component summary; MCS – mental component summary; TMD – total mood distribution.  Percent change in EQ-5D-5L Index score (IS) change was calculated as follows due to the possibility of negative scores:  $100*\left( \frac{{(IS}_{3m}- {IS}_{Baseline})}{Abs({IS}_{Baseline})} \right)$  For all other instruments, percent change was calculated as follows where score is the outcome measured by the instrument:  $100*\left( \frac{{(Score}_{3m}- {Score}_{Baseline})}{{Score}_{Baseline}} \right)$ | | | |

**References cited in the Supplement**

1. Vallejo R, Chakravarthy K, Will A, Trutnau K, Dinsmoor D. A New Direction for Closed-Loop Spinal Cord Stimulation: Combining Contemporary Therapy Paradigms with Evoked Compound Action Potential Sensing. *J Pain Res*. 2021;14:3909-3918. doi:10.2147/JPR.S344568

2. Grill WM, Mortimer JT. Electrical properties of implant encapsulation tissue. *Ann Biomed Eng*. Jan-Feb 1994;22(1):23-33. doi:10.1007/BF02368219

3. Kozai TD, Jaquins-Gerstl AS, Vazquez AL, Michael AC, Cui XT. Brain tissue responses to neural implants impact signal sensitivity and intervention strategies. *ACS Chem Neurosci*. Jan 21 2015;6(1):48-67. doi:10.1021/cn500256e

4. Cedeno DL. Post-Operative Changes of Chronically Recorded ECAP Latency in a Rodent Pain Model and Effects of ECAP Estimation Methods. Annual Meeting of the North American Neuromodulation Society; Las Vegas, NV. Jan 18-21, 2024.

5. Will A, Fishman M, Schultz D, et al. Improvements in Therapy Experience With Evoked Compound Action Potential Controlled, Closed-Loop Spinal Cord Stimulation-Primary Outcome of the ECHO-MAC Randomized Clinical Trial. *J Pain*. Nov 2024;25(11):104646. doi:10.1016/j.jpain.2024.104646
